# Standardizing pharmacology education in a systems-based curriculum: a longitudinal, multicampus innovations model

**DOI:** 10.3389/fphar.2026.1911082

**Published:** 2026-08-26

**Authors:** Jennelle Durnett Richardson, Angelika Martin, Lynn Roy, Brian Henriksen, Deron Herr, Caophi Nguyen, Charles N. Rudick, Brett Zimmerman, Larry Kilinski, Jonathan Guerrero

**Affiliations:** 1 Department of Biochemistry, Molecular Biology, and Pharmacology, Indiana University School of Medicine, Indianapolis, IN, United States; 2 Department of Biochemistry, Molecular Biology, and Pharmacology, Indiana University School of Medicine, Fort Wayne, IN, United States; 3 Department of Biochemistry, Molecular Biology, and Pharmacology, Indiana University School of Medicine, South Bend, IN, United States; 4 Department of Biochemistry, Molecular Biology, and Pharmacology, Indiana University School of Medicine, Terre Haute, IN, United States; 5 Department of Biochemistry, Molecular Biology, and Pharmacology, Indiana University School of Medicine, West Lafayette, IN, United States; 6 Department of Biochemistry, Molecular Biology, and Pharmacology, Indiana University School of Medicine, Bloomington, IN, United States; 7 Department of Biochemistry, Molecular Biology, and Pharmacology, Indiana University School of Medicine, Muncie, IN, United States; 8 Department of Anesthesia, Indiana University School of Medicine, Evansville, IN, United States; 9 Department of Biochemistry, Molecular Biology, and Pharmacology, Indiana University School of Medicine, Gary, IN, United States

**Keywords:** active learning, assessment, distributed campus, integrated curriculum, longitudinal integration, medical education, pharmacology education, spiral curriculum

## Abstract

Large, distributed medical schools face significant challenges in delivering consistent, high quality pharmacology instruction. Prior to 2016, the Indiana University School of Medicine (IUSM) delivered pharmacology as a traditional, standalone course at each of its nine campuses, resulting in variability in instructional approaches, assessments, and student experience. A major curriculum redesign provided the opportunity to re-envision pharmacology education within an integrated, organ system-based model. Pharmacology content is now intentionally embedded across seven organ system-based pre-clerkship courses, supported by a statewide pharmacology faculty team and a newly established Pharmacology Discipline Consultant. A total of 84 pharmacology contact hours were preserved, with 37 h of standardized recorded lectures introducing foundational concepts and 47 h of small-group active-learning sessions emphasizing application and integration. Content sequencing now follows a spiral design to introduce, revisit, and reinforce key drugs and concepts across systems. Assessment practices are unified statewide using the National Board of Medical Examiners (NBME) item writing principles, centrally coordinated item review, and iterative quality improvement based on student feedback and psychometric performance. All pharmacology content and assessments are delivered identically across campuses to ∼375 students statewide. Recorded lectures provide uniformity in presentation and expectations, while campus-based active learning enables local faculty to facilitate case-based reasoning and integration across courses. Structured facilitator guides support faculty in synchronized delivery of the pharmacology curriculum. A statewide review process maintains alignment of content and reinforces consistency over time. This model preserves total pharmacology instructional time despite distributing content across multiple courses, maintaining coherence of the pharmacology curriculum through intentional sequencing, and establishes consistent assessment quality across all campuses. Student satisfaction with pharmacology instruction remains high, and learners report confidence in the alignment between instruction and assessment. Embedding a longitudinal pharmacology curriculum within an organ system-based framework is feasible and effective when supported by centralized coordination, standardization of foundational content, and faculty development. This innovation provides a replicable model for maintaining a high-quality, active-learning rich pre-clerkship pharmacology curriculum distributed across courses and campuses.

## 1 Introduction

**FIGURE 1:**
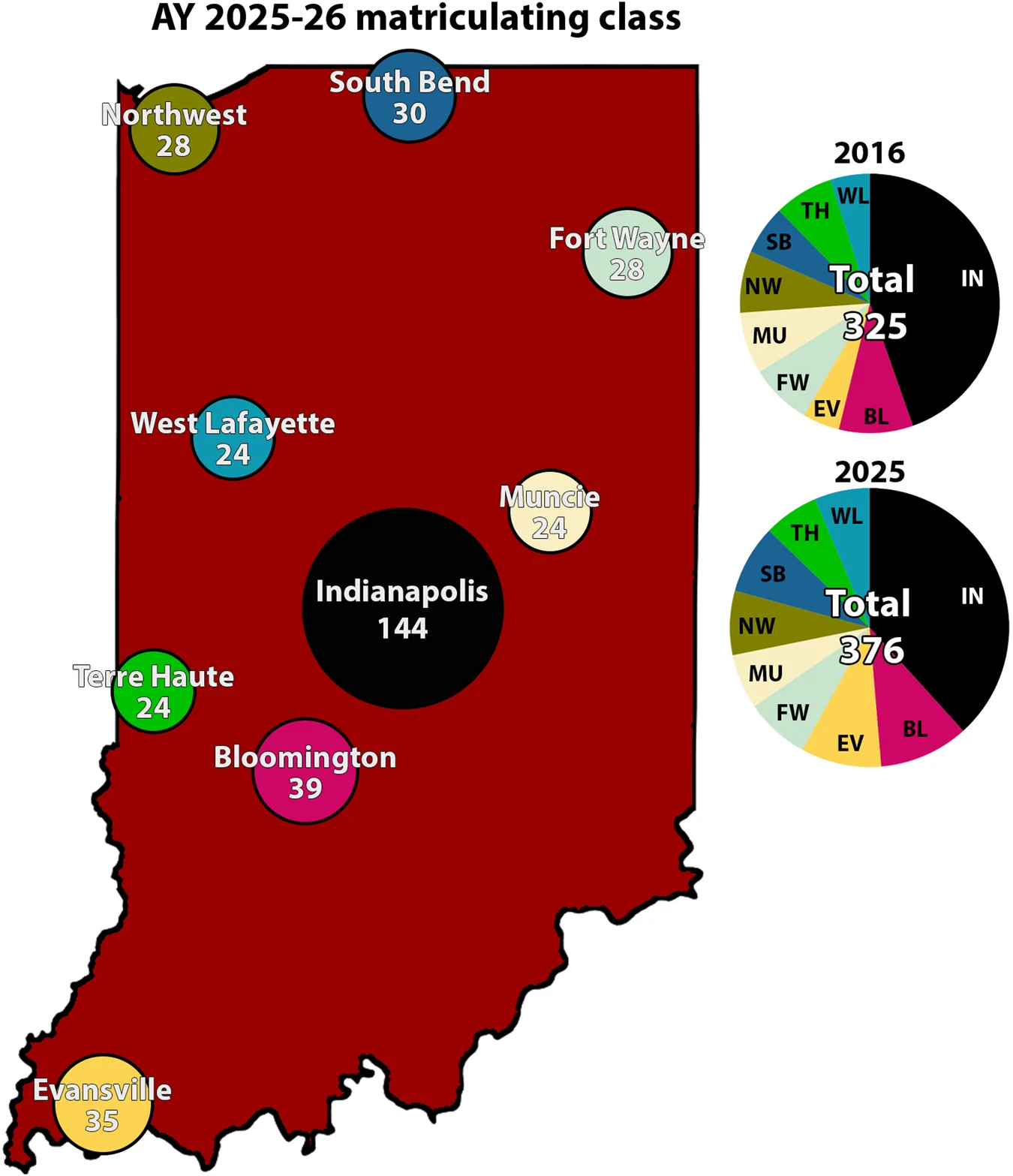
Map of Indiana illustrating the nine Indiana University School of Medicine (IUSM) regional campuses, annotated with matriculating student numbers for the 2025–2026 academic year. Pie charts compare the distribution of the matriculating class across campuses in 2016, before curricular reform, with the current distribution in 2025.

Indiana University School of Medicine (IUSM) is the largest allopathic medical school in the United States enrolling approximately 375 students annually across nine campuses with cohort size varying between 24 and 144 students per campus (Figure 1).

Prior to 2016, pharmacology instruction during the pre-clerkship phase followed a traditional discipline-based model rooted in Flexner’s 1910 curriculum framework (Flexner, 1910), delivered largely through didactic lectures with notable variability across campuses (Table 1). Although statewide expectations mandated common learning objectives and an identical final assessment (the National Board of Medical Examiners (NBME) pharmacology shelf examination), individual campuses differed in duration (5-week blocks to full-year formats), contact hours (80–99 h), pedagogical approach (lecture-based, problem-based learning, team-based learning), exam number (1–9), and grade distribution (Honors, High Pass, Pass, and Fail). This fragmentation created inconsistencies in curricular delivery and student experiences across the geographically distributed student cohorts.

**TABLE 1:** Comparison of pharmacology curriculum characteristics before and after statewide curricular redesign.

Curricular characteristic	Discipline-based pharmacology curriculum	Organ system-based pharmacology curriculum
Curriculum development	Campus course directors	Statewide pharmacology team
Campus comparability	Shared course learning objectives and final exam	Standardized learning objectives, lectures, active learning materials, facilitator guides, formative and summative assessments
Course length	Large variability across campuses: 5 weeks to full year	Longitudinal across seven pre-clerkship courses (∼14 months)
Contact hours	80–99 h	84 h
Number of courses	1	7
Teaching methods	Seven campuses relied primarily on in-person lectures One campus was fully dedicated to problem-based learning (PBL) One campus heavily utilized team-based learning (TBL)	Standardized recorded lectures and case-based active learning sessions
Number of exams	Intercampus variability: 1–9	24 exams: 1 exam dedicated exclusively to pharmacology 23 multidisciplinary exams that include pharmacology questions alongside other subjects
Assessment development	Campus-developed with common final exam	Statewide assessments using NBME item-writing guidelines, centralized review, and psychometric analysis
Grade structure	Honors, high pass, pass	Pass, fail
Campus pharmacology faculty	One primary pharmacology educator per campus, typically a basic scientist or pharmacist	One primary pharmacology educator per campus (basic scientist, pharmacist, or physician) participating in the statewide pharmacology team
Faculty development	Local	Statewide facilitator guides and ongoing faculty development opportunities

Prior to the implementation of the integrated curriculum, each of the nine campuses independently developed and delivered its own pharmacology curriculum while sharing common Course Objectives and the NBME Pharmacology Shelf Exam. Following implementation, all campuses adopted standardized instructional materials, active learning resources, facilitator guides, assessments, and statewide curricular oversight.

Concurrent with an upcoming accreditation review and a growing body of literature favoring integrated curricula and active learning (Brauer and Ferguson, 2015; Fasinu and Wilborn, 2024), IUSM undertook a major pre-clerkship redesign beginning in 2016. The new model replaced discipline-based courses with organ system-based courses, limited didactic teaching to fewer than 50% of contact hours, expanded active learning, offered earlier clinical exposures, and required synchronized statewide delivery.

During early planning, faculty raised concerns that pharmacology content, when distributed across seven organ system-based courses, might become diluted or overshadowed by clinical material. To address this, IUSM appointed a Pharmacology Discipline Consultant to develop and coordinate content, ensure consistency, and maintain the rigor of foundational pharmacology instruction.

The following sections describe how the largest allopathic medical school in the United States successfully navigated the challenges of integrating pharmacology into an organ system-based curriculum across nine campuses, outlining both the obstacles encountered and the coordinated strategies that enabled the development of a fully standardized longitudinal pharmacology curriculum with nearly a decade of demonstrated success.

## 2 Methods

### 2.1 Challenges in integration: discipline integrity, distributed campuses, faculty adoption, learning environment, and pedagogical format

Transitioning pharmacology from a standalone discipline-based course into an integrated organ system-based curriculum introduced several anticipated and emergent challenges.

#### 2.1.1 Maintaining pharmacology discipline identity

**FIGURE 2:**
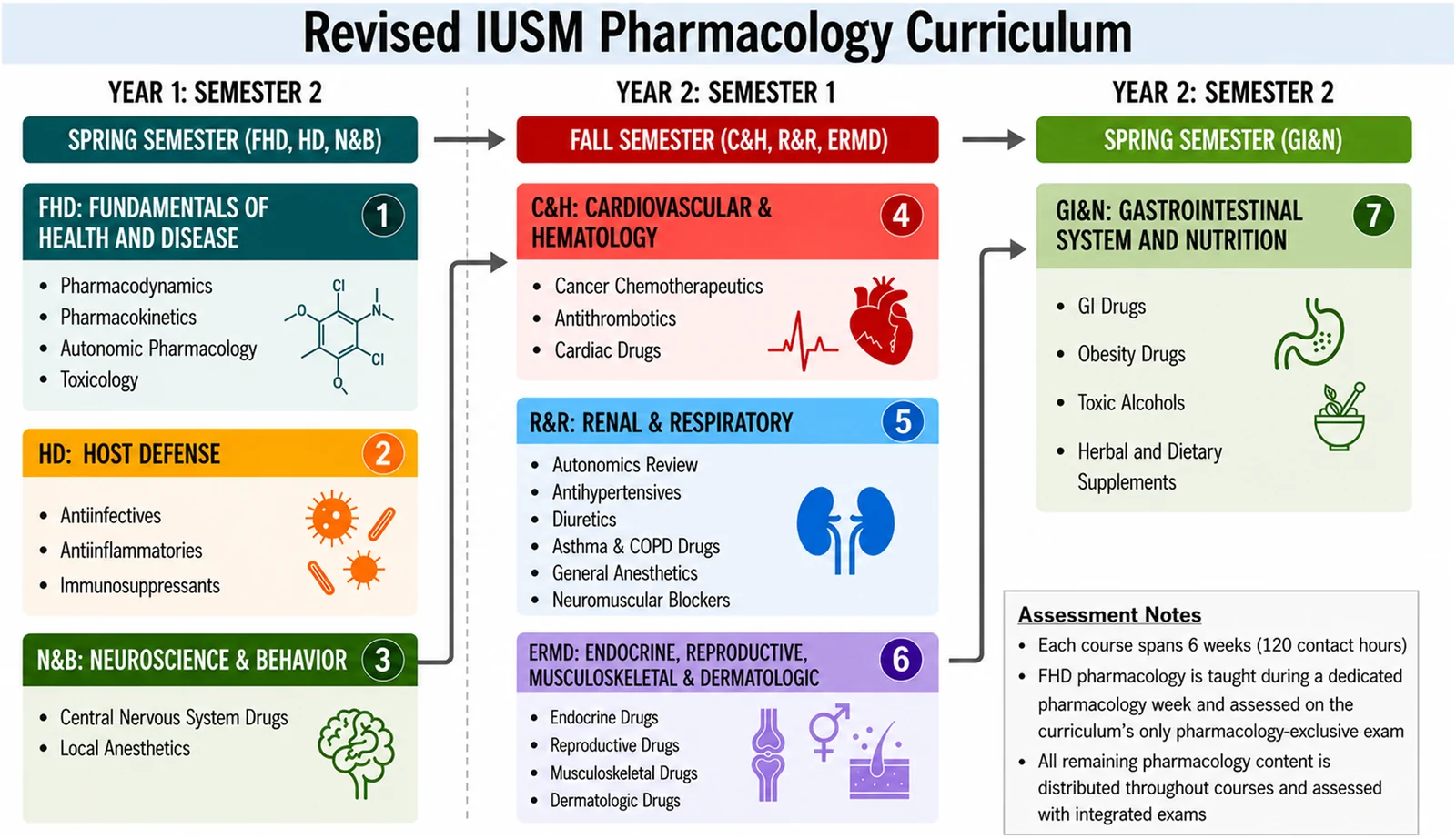
Sequencing of the revised pharmacology curriculum. Pharmacology is integrated across seven pre-clerkship courses. Each course spans 6 weeks. In Fundamentals of Health and Disease (FHD), pharmacology is taught during a dedicated pharmacology week and assessed on a dedicated pharmacology examination. In all subsequent courses, pharmacology content is distributed throughout the course alongside non-pharmacology content and assessed as part of integrated examinations.

A significant concern involved maintaining the disciplinary identity and integrity of pharmacology once it was no longer delivered as a standalone course but instead embedded within seven organ system-based courses during the pre-clerkship years (Figure 2). Faculty worried that pharmacology content could be overshadowed by broader clinical concepts within the organ system-based blocks. Because each course was administered by a multidisciplinary Course Management Team (CMT), many of whom lacked pharmacology expertise, there was a real possibility that essential content might be deprioritized or inconsistently represented across courses and campuses, potentially reducing dedicated contact hours, diminishing the number of pharmacology-focused assessment items, and ultimately compromising student knowledge and competence in this essential domain.

#### 2.1.2 Ensuring statewide consistency

Ensuring uniformity across IUSM’s nine campuses also posed a significant structural challenge. Under the discipline-based model, instructors at each site created their own lectures, cases, and assessments, leading to considerable variation in content emphasis and instructional approach. Transitioning to the integrated curriculum required replacing this locally driven model with a statewide system in which all campuses utilize identical, centrally developed materials. This shift was essential to maintaining curricular coherence and ensuring that students, regardless of campus assignment, received equivalent exposure to pharmacology content.

To achieve and maintain comparability across each of its nine campuses, each course formed a CMT consisting of at least one representative from each of IUSM’s nine campuses. Many of those CMT members did not have expertise in pharmacology, and a few of the CMTs had no pharmacology representation. The CMTs developed course learning objectives, directed development of content and assessments, and were responsible for the delivery of the course on their respective campus. The multi-campus faculty structure and multidisciplinary composition of the course leadership introduced a risk of inconsistency in the depth, pacing, and emphasis of pharmacology across the geographically separated cohorts. In teams lacking pharmacology representation, there was concern that pharmacology-specific learning objectives and instructional practices might not be adequately incorporated. Even with shared statewide expectations, the implementation of integrated content risked diverging over time without a coordinated mechanism to align teaching materials, reinforce standards, and ensure accountability.

#### 2.1.3 Faculty adaptation to new pedagogies

The transition in pedagogy further complicated integration efforts. Moving from predominantly in-person lectures to asynchronous recorded content (representing fewer than 50% of contact hours) removed the immediate verbal and nonverbal feedback instructors traditionally relied on to gauge student comprehension. This loss of interaction introduced uncertainty about how well students were absorbing foundational material and hindered faculty’s ability to adjust explanations in real time.

At the same time, active-learning formats, central to the redesigned curriculum and representing more than 50% of contact hours, were unfamiliar to many pharmacology instructors. Facilitating case discussions, prompting mechanistic reasoning, and managing small group dynamics represented skills that required dedicated faculty development and practice.

#### 2.1.4 Managing cognitive load and sequencing

Coordination across disciplines and campuses also created logistical complexities. Integrated courses required tight alignment between pharmacology, physiology, pathology, and clinical medicine, yet synchronous delivery of active-learning sessions across all campuses meant that timing misalignments could easily occur due to differences in local faculty availability. When foundational physiology or pathology content preceded (or followed) pharmacology sessions in ways that differed from the intended sequence, students occasionally experienced brief gaps or redundancies in their learning.

Cognitive load considerations presented another challenge. The transition to an integrated model required careful sequencing of pharmacology to avoid overwhelming students while still ensuring sufficient reinforcement across courses. Revisiting drugs in multiple contexts supports deeper learning, yet without a transparent spiral framework, learners occasionally perceived repeated exposure as unnecessary overlap rather than intentional pedagogical scaffolding. Balancing reinforcement with novelty, minimizing redundancy, and communicating the rationale for repeated encounters requires ongoing coordination and refinement.

Together, these challenges shaped the development of governance structures, pedagogical support, and sequencing decisions that ultimately guided the successful integration of pharmacology throughout the pre-clerkship organ system-based curriculum at IUSM.

### 2.2 Approach to integration: pedagogical principles, design, and implementation

#### 2.2.1 Statewide pharmacology team

While the formation of CMTs focused on campus representation rather than ensuring discipline representation, all nine campuses had a dedicated pharmacology faculty member involved in the delivery of pharmacology content. Most of these faculty were PhD basic scientists while a few had clinical experience as pharmacists, and one was an MD. These faculty formed the Statewide Pharmacology Team. One of the members took the role of the Pharmacology Discipline Consultant who served to coordinate the pharmacology efforts and represent pharmacology interests with each of the CMTs.

The Statewide Pharmacology Team met regularly to coordinate learning objectives, review and revise teaching materials, develop and review assessment items, and ensure coherent sequencing across the curriculum. Additionally, the team supported faculty development in active learning strategies.

The Pharmacology Discipline Consultant played a central coordinating role, overseeing content development, creation of recorded lectures and active-learning materials, harmonizing sequencing with other foundational sciences, leading statewide assessment processes, and facilitating statewide communication with faculty and learners. This hybrid model, a centralized design paired with distributed delivery, ensured that all students received equivalent instruction while also benefiting from local faculty expertise.

#### 2.2.2 Guiding pedagogical principles

The integration of pharmacology into the organ system-based curriculum at IUSM was guided by a set of intentional pedagogical and structural principles designed to preserve disciplinary rigor while supporting the broader goals of the curricular reform. From the outset, the curriculum redesign emphasized centralized coordination of foundational content, predictable instructional structure, and the incorporation of active-learning strategies shown to support durable understanding in the health sciences (Kiviniemi, 2014). The shift from a discipline-based course to a longitudinally integrated framework required reimagining how pharmacology concepts were introduced, reinforced, and applied across seven pre-clerkship organ system-based courses. This multiyear process included detailed curriculum mapping, collaborative content development, and implementation planning conducted by the statewide pharmacology faculty team and overseen by the Pharmacology Discipline Consultant.

#### 2.2.3 Learning objectives

Pharmacology learning objectives were centralized, course-mapped, and shared across all campuses. These objectives explicitly aligned with the associated organ system-based course objectives and were written to enable foundational pharmacology knowledge acquisition and application to clinical scenarios. Learning objectives were also sequenced according to a spiral curriculum. Core drug classes and mechanisms were introduced early (e.g., autonomic pharmacology), then revisited and expanded within relevant organ blocks (e.g., cardiovascular and neurology courses), balancing spaced reinforcement (Cepeda et al., 2008) with intentional minimal redundancy allowing students to progressively expand knowledge without becoming overwhelmed.

#### 2.2.4 Centralized recorded lectures

A crucial element of the integration strategy was the development of standardized, recorded lectures for use across all nine campuses. During the early phase of the curricular reform, uniform slide decks were created and shared with faculty statewide for live delivery at each campus. These live lectures were recorded and accessible asynchronously to all students statewide. This led to a substantial number of students watching multiple postings of the same lecture content delivered at different campuses to avoid missing a critical piece of information that may have been delivered orally. Thus, it was decided to replace live lectures with a single statewide recording for each lecture such that a single recorded lecture was utilized by students at all campuses. The uniform lectures are distributed among the Statewide Pharmacology Team for recording so that all pharmacology faculty contribute to the recorded pharmacology curriculum. The transition to recorded delivery ensures that the foundational pharmacology content is presented with consistent depth, organization, and terminology, thereby promoting consistency and reducing variability in student learning experiences. The distribution of recording responsibilities across the Statewide Pharmacology Team also reinforces the one-school nature of the curriculum. Additionally, to reduce extraneous cognitive load and reinforce learning patterns, each lecture follows a uniform structure and uses consistent visual formatting. The recorded format also facilitates efficient annual updates, ensuring that content remains current across all campuses.

To address the lack of verbal and non-verbal feedback typically obtained during in-person lectures, students are encouraged to provide written feedback on asynchronous lectures via Qualtrics surveys linked to each lecture. They are also encouraged to use the asynchronous Q&A community platform InScribe to raise questions or clarify points of confusion, which are promptly addressed by pharmacology faculty. This feedback informs iterative improvement to lecture materials and helps identify misconceptions that can be addressed during active-learning sessions.

The longitudinal design follows a spiral sequencing model (Cepeda et al., 2008) in which core concepts are introduced early and then revisited in later organ system-based courses with increasing clinical complexity. For example, propranolol is first introduced when discussing autonomic pharmacology, revisited several weeks later in the context of migraine management, and reinforced a few months later in the cardiovascular block. This intentional revisiting of pharmacology content allows students to reinforce foundational principles and extend their understanding as they encounter related physiology and pathology.

#### 2.2.5 Active learning model

Complementing the recorded lectures, the curriculum placed a strong emphasis on non-didactic active learning sessions delivered locally at each campus. These active learning sessions are based on a flipped classroom model and small group learning (Kennedy, 2018; Ballouk et al., 2022; Westerdahl et al., 2022). Learners in groups of six to eight students work collaboratively to apply fundamental knowledge gained through recorded lectures in application-focused exercises, including clinical cases, mechanism-based reasoning prompts, and structured analysis of therapeutic decisions. The sessions are designed for students to work through the exercises relying on the knowledge of their group in a peer-assisted model. A single pharmacology facilitator is then able to clarify, reinforce, and expand knowledge during the full-class discussion portion of each session. To support student engagement and the ability of facilitators to identify gaps in knowledge, particularly at our largest campus with 36 groups of students and a single facilitator, instructional technologies such as audience response systems are utilized to quickly gauge the response of all groups. This allows the facilitator to better balance the full-class discussion time focusing on concepts needing clarification and reinforcing and expanding other areas. This peer-assisted learning approach is generally regarded as more student-centered, with educators functioning more as a facilitator of learning rather than an instructor (Gupta et al., 2014; Vora and Shah, 2015; Chiranjeevi et al., 2022).

The Statewide Pharmacology Team has developed detailed guides outlining key concepts, anticipated misconceptions, discussion pathways, and recommended depth of coverage. This structure allows faculty to connect pharmacology content across courses, highlight mechanistic relationships, and reinforce learning in a manner tailored to student needs while maintaining statewide alignment.

#### 2.2.6 Assessment integration

Assessment integration represents another critical component of this approach. Students receive both formative and summative feedback to evaluate the effectiveness of their study strategies and monitor their understanding of the material. Formative assessments are embedded within lecture modules, providing opportunities for students to assess their foundational pharmacology knowledge before engaging in active-learning sessions. These brief knowledge checks help identify gaps in understanding early, enabling students to arrive better prepared for application-based activities. In addition, the exercises completed during the active learning sessions function as formative assessments, allowing students to further refine their understanding and address remaining knowledge gaps prior to summative evaluation.

Summative assessments are designed to measure higher-order thinking and the application of pharmacology concepts introduced in the recorded lectures and reinforced through active learning activities. Rather than focusing solely on recall, these assessments evaluate students’ ability to integrate, analyze, and apply pharmacology principles in clinically relevant contexts. Each block exam includes NBME-style pharmacology questions written using established item writing guidelines. All assessment items undergo a rigorous process of pre-exam peer review and post-exam psychometric analysis including difficulty, discrimination, and option performance to ensure quality and alignment with learning objectives. Students are also able to provide feedback on exam questions through the exam software. This facilitates identification of unclear question wording or misalignment between assessment items and presented content. This iterative cycle strengthens the reliability and validity of the question bank over time. Learners are given immediate access to rationales for all incorrect responses upon conclusion of their examination. This timely feedback supports rapid clarification of misconceptions, encourages self-explanation, and guides efficient self-directed review. In addition to block exams, every organ system-based course concludes with a cumulative customized NBME final exam with pharmacology questions selected and curated by the Pharmacology Discipline Consultant.

#### 2.2.7 Contact hours and longitudinal structure

Finally, the curriculum preserved dedicated pharmacology instructional time while distributing content across seven integrated courses. The model maintains 84 total contact hours, consisting of 37 h of recorded lectures and 47 h of active learning activities (∼56% of total curricular hours). To maintain curricular coherence over time, the Statewide Pharmacology Team conducts annual reviews of contact hours, session sequencing, content coverage, and assessment weighting to ensure continued alignment and prevent curriculum drift.

**FIGURE 3:**
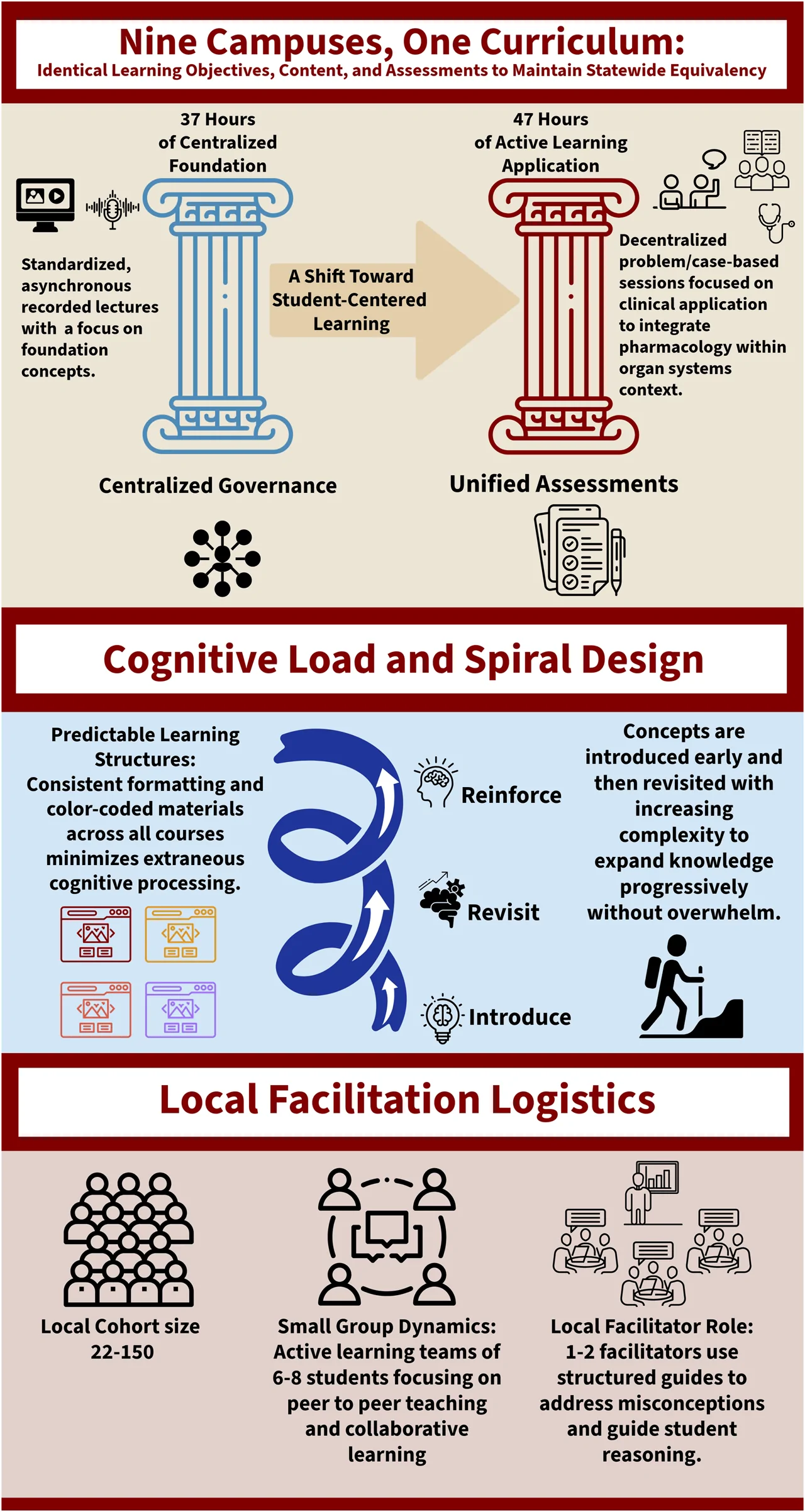
Overview of the multi-campus pharmacology curriculum framework. Standardized foundational content and unified assessments allow maintenance of statewide equivalency across the nine campuses. Consistent course structures reduce cognitive load, and the spiral design allows for progressive reinforcement of concepts. Active learning sessions using case- and problem-based learning implemented locally through small group-facilitation emphasize collaborative learning and clinical application.

Through a coordinated strategy that integrates centralized content development, standardized instructional methods, spiral curricular sequencing, faculty development, and robust governance structures, IUSM has established a cohesive and sustainable longitudinal pharmacology curriculum. This approach preserves the integrity and visibility of the discipline while leveraging the advantages of an integrated, organ system-based curriculum (Figure 3).

## 3 Results

### 3.1 System-level outcomes

The integrated pharmacology curriculum resulted in demonstrably reliable statewide implementation across all nine IUSM campuses. Standardized recorded lectures, shared active-learning exercises, and synchronized scheduling ensured that students received equivalent instructional and assessment experiences regardless of geographic location. This consistency represented a substantial improvement over the pre-2016 curricular model, in which locally developed materials often produced variability in pharmacology exposure and depth.

Standardization of the pharmacology curriculum had the unintended benefit of providing an instructional framework for new faculty. Instructors from basic science disciplines typically are not formally trained in pedagogy and may have limited clinical experience. This presents a challenge in integrating clinical relevance into the instruction of basic science principles (Greene et al., 2018; Dominguez and Zumwalt, 2020). The standardized curriculum and detailed facilitator guides support consistent implementation of the active learning sessions across all nine campuses. Evidence of this consistency is reflected in student performance on the only examination within the curriculum dedicated exclusively to pharmacology (Table 2). For the 2025–2026 academic year, across the nine campuses, the mean exam score was 84% with a standard deviation of only 2% indicating minimal variation in performance between campuses. In contrast, the standard deviation of student scores within individual campuses ranged from 8% to 12%, indicating that variability among students was substantially greater than variability across campuses. These findings suggest that the statewide pharmacology curriculum is being implemented consistently and achieving comparable education outcomes regardless of campus location.

**TABLE 2:** Campus performances on the pharmacology-exclusive exam during the 2025–2026 academic year.

Campus	n	Average (%)	Standard deviation (%)
Campus 1	28	81.7	9.1
Campus 2	24	82.7	8.4
Campus 3	24	82.9	9.7
Campus 4	28	83.6	8.4
Campus 5	34	84.2	9.6
Campus 6	30	84.2	10.8
Campus 7	24	85.5	11.6
Campus 8	39	86.0	9.4
Campus 9	140	88.1	9.3
Statewide	371	84.3	2.0

Campus performance on the only pre-clerkship exam devoted exclusively to pharmacology during the 2025–2026 academic year. Students across different campuses perform very similarly on average with a standard deviation of approximately 2%, but individual student scores within the same campus vary widely from 8.4% to 11.6%. Out of 24 pre-clerkship exams that include pharmacology, this is the only exam composed exclusively of pharmacology questions.

Another key outcome was the significant increase in faculty collaboration across the statewide system. The integrated model encouraged pharmacology instructors from all campuses to join forces regularly on content development, session design, sequencing, longitudinal balance, and annual curriculum review. These ongoing collaborations fostered a stronger sense of shared ownership of the pharmacology curriculum and created a robust community of practice. Faculty engagement expanded beyond content planning to include peer review of assessments and discussions on best practices in facilitation. Pharmacology faculty are routinely recognized for excellence in teaching through school and student-led teaching awards.

The quality of block exam questions similarly improved through systemic adoption of NBME-guided item writing practices and a structured statewide review process. Following each examination, items underwent comprehensive psychometric analysis including evaluation of difficulty, discrimination, and option performance which informed iterative revisions and contributed to more reliable and valid assessments. The use of customized NBME final examinations at the end of each organ system-based course further strengthened alignment with national standards and ensured that the curriculum prepared students for the cognitive demands and item formats encountered on clerkship NBME shelf exams and national licensing exams.

### 3.2 Preservation of instructional rigor and pedagogical impact

**FIGURE 4:**
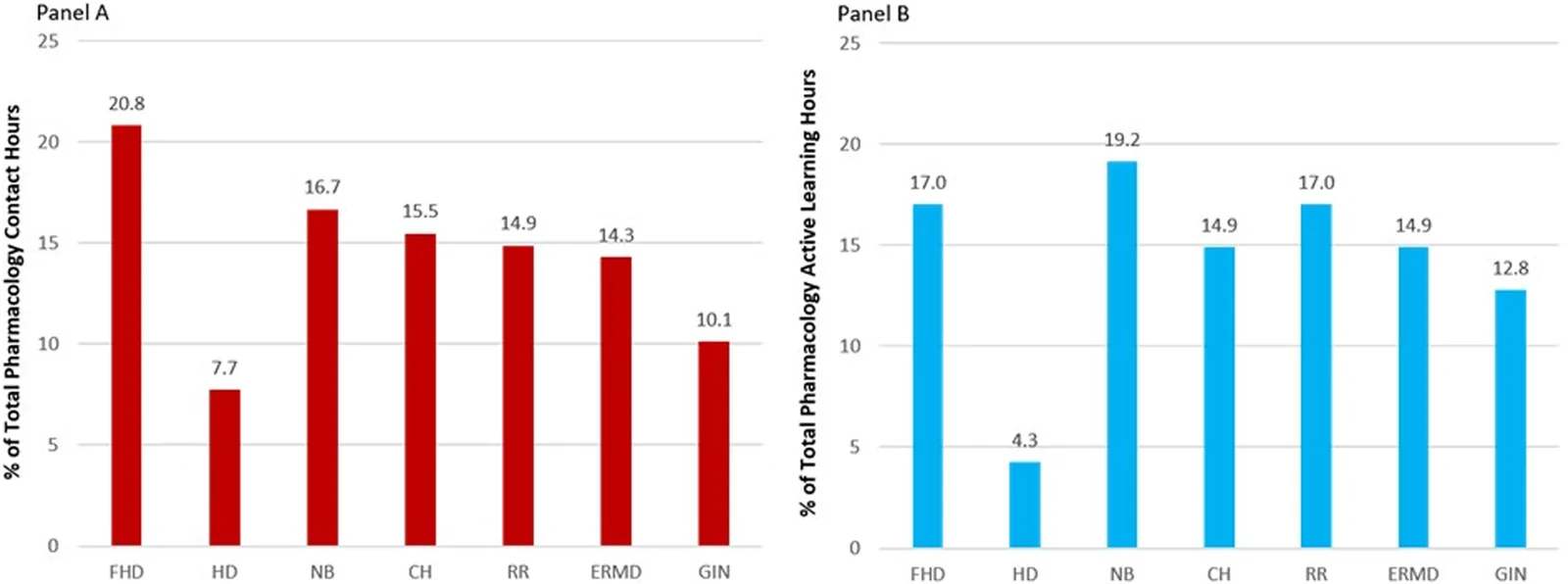
Distribution of pharmacology curriculum across courses in terms of the percentage of total pharmacology curricular contact hours and percentage of pharmacology active learning contact hours in each course. FHD: Fundamentals of Health and Disease; HD: Host Defense, NB: Neuroscience and Behavior; CH: Cardiovascular and Hematology; RR: Renal and Respiratory; ERMD: Endocrine, Reproductive, Musculoskeletal, and Dermatologic; GIN: Gastrointestinal System and Nutrition.

Due to intercampus variability in the pre-integration discipline-based curriculum, totals are reported as ranges in the following descriptive comparison while using single numbers for the post-integration organ-based curriculum. Post-integration, pharmacology content and active-learning sessions were dispersed fairly evenly across seven courses (Figure 4) with total pharmacology instruction ranging from 6.5 to 17.5 h per course (Supplementary Table S1), of which 2–8 h were dedicated to active learning sessions. Pharmacology maintained total contact hours within the historic range (84 h post-integration vs. 80–99 h pre-integration). Active-learning hours increased substantially from 14 to 37 h pre-integration to 47 h (55.95%) post-integration, emphasizing application-based learning and limiting didactic time to 37 h (44.05%).

Performance on pharmacology questions included in the customized NBME final examinations provides an additional objective indicator of students’ pharmacology knowledge (Table 3). IUSM students performed at or above the national NBME reference group on pharmacology questions, exceeding the national NBME reference group on five of the seven customized NBME final examinations. Across the seven exams, an average of 83% of IUSM students answered pharmacology questions correctly, compared with 79% for the national NBME reference group. Because multiple changes accompanied the transition from the discipline-based to the organ system-based curriculum, student performance cannot be directly compared before and after curricular implementation. Nevertheless, these findings are consistent with the organ system-based curriculum providing students with a strong foundation in pharmacology.

**TABLE 3:** Performance on pharmacology questions on course NBME Final exams.

Course	IUSM average	NBME average
FHD	0.83	0.80
HD	0.82	0.84
NB	0.76	0.77
CH	0.85	0.79
RR	0.84	0.78
ERMD	0.80	0.68
GIN	0.92	0.84
Average	0.83	0.79

Average item difficulty for pharmacology questions on customized NBME final examinations between IUSM students and the national NBME reference group. FHD: Fundamentals of Health and Disease; HD: Host Defense, NB: Neuroscience and Behavior; CH: Cardiovascular and Hematology; RR: Renal and Respiratory; ERMD: Endocrine, Reproductive, Musculoskeletal, and Dermatologic; GIN: Gastrointestinal System and Nutrition.

### 3.3 Student satisfaction (Strategic Student Survey (S3) 2021–2025)

The integrated curricular approach not only enhanced curricular coherence but also reduced student cognitive load by establishing predictable structure across courses. The consistent organization of recorded lectures, uniform formatting of learning materials, and stable pattern of active-learning sessions enabled students to develop efficient study routines and anticipate instructional expectations. The spiral sequencing of pharmacology content provided purposeful reinforcement without unnecessary redundancy, supporting deeper integration of concepts across organ systems. Together, these improvements created a more streamlined and cognitively manageable learning environment as indicated from the Strategic Student Survey (S3). The S3 at IUSM is an annual, internal survey designed to gather actionable feedback from medical students across all nine campuses regarding their educational experience. In 2021, questions regarding satisfaction with pre-clerkship educational experience were added for each discipline. Results indicate high levels of student satisfaction for both first year (MS1) and second year (MS2) cohorts (90%–96%), with only modest year-to-year variation (Table 4).

**TABLE 4:** Strategic Student Survey: student satisfaction with the pharmacology educational experiences.

Year (response rate MS1, MS2)	% Satisfaction	
	MS1	MS2
2021 (50%, 57%)	93%	90%
2022 (82%, 59%)	91%	93%
2023 (84%, 81%)	95%	90%
2024 (86%, 81%)	95%	93%
2025 (77%, 79%)	94%	96%

The data displays the percentage of students who chose Very Satisfied or Satisfied regarding the overall quality of the pre-clerkship phase of the curriculum. The remaining respondents selected Dissatisfied, Very Dissatisfied, or Not Applicable.

### 3.4 National performance (Graduation Questionnaire (GQ) 2020–2025)

**FIGURE 5:**
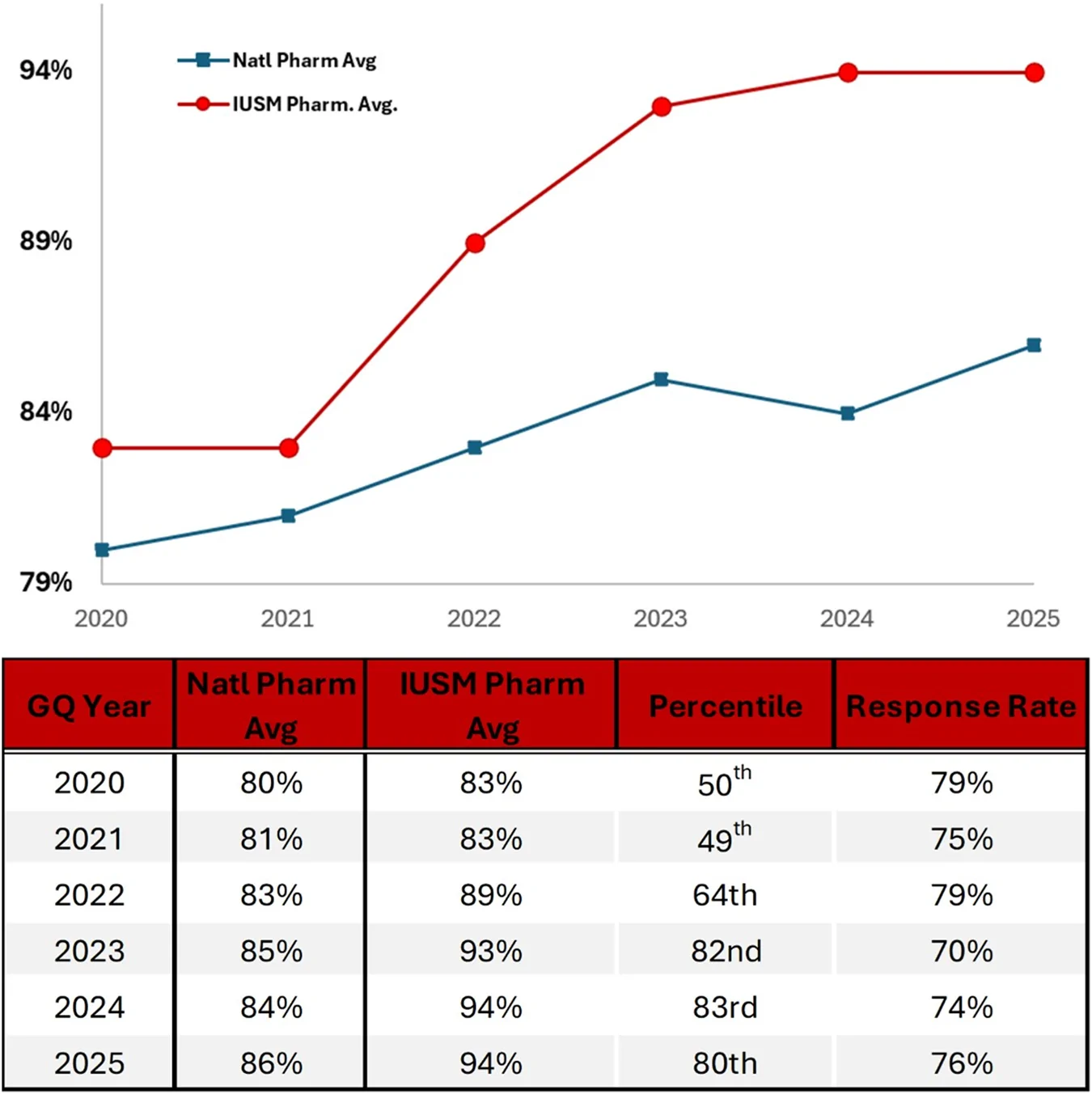
Rating of pharmacology preparation for clerkships as good or excellent as reported by the AAMC GQ.

Students in the first year of the integrated curriculum completed the Association of American Medical Colleges (AAMC) Graduation Questionnaire (GQ) in 2020 (Figure 5). The percentage of IUSM graduating students rating IUSM’s pharmacology preparation for clerkships as Good or Excellent on the GQ increased from 83% to 94% across six cohorts reflecting a strong linear trend of +2.63 percentage points per year (*R* ^2^ = 0.88). During the same period, the national average rose more modestly from 80% to 86% (+1.17 pp/year; *R* ^2^ = 0.90), resulting in a widening institutional advantage of +1.46 pp/year and an average gap of +6.17 points across the 5-year span.

IUSM’s percentile rank improved from approximately the 50th percentile to a sustained position above the 80th percentile in recent years. These gains have stabilized across the most recent cohorts, indicating durable, rather than transient, improvement. Overall, pharmacology preparation is now consistently rated in the 93%–94% range, outperforming national benchmarks and indicating that students perceive that the integrated curriculum provides strong preparation for pharmacology in the clinical setting.

Taken together with pre-clerkship satisfaction levels that have remained above 90% annually in both MS1 and MS2 cohorts, these outcomes indicate a broadly stable curriculum, a supportive learning environment, and strong alignment with student expectations and educational needs.

## 4 Discussion

The transition from a traditional discipline-based pharmacology curriculum to an organ-based integrated model at IUSM did not diminish the rigor, breadth, depth, or time devoted to pharmacology instruction. Despite concerns regarding potential erosion of disciplinary identity during integration, our findings demonstrate that total pharmacology contact hours were fully preserved following the curricular reform. This stability in instructional time suggests that integration, when intentionally designed, can maintain essential foundational science content while supporting broader curricular coherence.

A notable outcome of the redesigned curriculum was the substantial expansion of active-learning pedagogy, both in absolute hours and as a proportion of total instructional time. Across the seven organ-system courses, most allocated 50% or more of pharmacology contact hours to active-learning modalities indicating that the increase was broad-based rather than isolated to a subset of courses. Although this distribution was not fully uniform, the overall pattern reflects a meaningful and systemic pedagogical shift aligned with evidence favoring active engagement for promoting durable learning (Firman et al., 2025; Freeman et al., 2014). The variability between courses reflects differences in course design and topic-specific opportunities for application. Content distribution and exposure is an area that is reviewed annually to determine opportunities for greater engagement.

Importantly, these structural and pedagogical changes were accompanied by high and sustained levels of student satisfaction with the pharmacology learning experience. Satisfaction ratings remained consistently above 90% across both MS1 and MS2 cohorts during the 2021–2025 period, indicating that students perceived the integrated curriculum as supportive, coherent, and effective. This alignment between curricular reform and students’ subjective experience strengthens the argument that integration, when implemented with attention to instructional quality, enhances rather than detracts from learners’ perceptions of preparedness and relevance. Furthermore, we attribute these high satisfaction scores also to the commitment of IUSM to employ basic science educators and give them the protected time required for engaging in scholarly teaching.

Student satisfaction with pharmacology is maintained through their final years of medical school. The percentage of graduating medical students who rated IUSM’s instruction in pharmacology as Good or Excellent has improved at a rate exceeding national trends, rising more quickly than the national average and stabilizing at consistently high values (93%–94%) in the most recent cohorts. Percentile rank data also demonstrate strong external standing, with IUSM scoring in the ∼80th percentile or higher in recent years. The combination of an accelerating gap between institutional and national means and stability of performance across the last three cohorts indicates that these improvements represent a sustained, structural advancement, not a transient or cohort-specific peak. The learner perceptions are complemented by objective assessment data, with IUSM students exceeding the national NBME reference group on pharmacology questions in five of the seven customized NBME final examinations. Taken together with the preserved contact hours and increased active-learning emphasis, these findings suggest that integration of pharmacology into organ system-based courses can maintain a rigorous pharmacology curriculum while providing a positive learning experience.

The redesigned curriculum also benefited from the intentional use of spiral sequencing, which strengthened reinforcement by allowing students to revisit core pharmacology concepts multiple times at progressively higher levels of complexity throughout the pre-clerkship curriculum. This repeated exposure supported deeper integration of foundational principles with emerging clinical applications and improved long-term retention. The positive effects of a spiral curriculum were demonstrated by Maltagliati et al. (Maltagliati et al., 2023), who evaluated a pre-clerkship model using spaced repetition and board-style questions to reinforce prior content across three medical school classes. They further emphasized that such curricula should be implemented flexibly, as rigid, mandatory review sessions, particularly in accelerated programs, added workload and stress, and were perceived negatively by students.

In parallel, the adoption of a predictable course structure enhanced student engagement and reduced cognitive load. By maintaining consistent patterns in session formats, learning expectations, and assessment design, the curriculum minimized unnecessary extraneous processing and enabled students to devote more cognitive resources to meaningfully engaging with the material. Together, these design features contributed to a more coherent learning experience that supported both conceptual mastery and efficient knowledge acquisition. Similar conclusions were drawn by Khurshid et al. (Khurshid et al., 2023) whose review of 75 studies demonstrated that pedagogical interventions such as scaffolding, intentional reduction of cognitive load, and active learner engagement were consistently more effective, enhancing students’ understanding of core pharmacology concepts and improving their satisfaction with learning, frequently resulting in higher assessment performance.

Overall, the findings support the conclusion that an integrated pharmacology curriculum can maintain disciplinary integrity while promoting increased engagement. The combination of preserved rigor, expanded active learning, high student satisfaction, and favorable national survey outcomes positions the integrated model as a robust and effective approach to foundational pharmacology education. Future work may explore how specific active-learning strategies or course-level structures contribute to these gains, as well as the downstream impact on clerkship readiness and prescribing competence.

In addition to its pedagogical improvements, the IUSM model demonstrates several structural strengths that make it particularly well-suited for large, distributed medical education systems. The curriculum was intentionally designed for scalability across nine campuses, supported by strong institutional governance, clear decision-making pathways, and shared accountability structures. This enabled consistent implementation of curricular standards without sacrificing local adaptability. A defining feature of the model is its hybrid instructional approach, in which centralized lectures ensure uniform delivery of foundational pharmacology content, while local delivery of active-learning sessions allows for direct interaction of students with pharmacology content experts who provide longitudinal content integration and support throughout the pre-clerkship phase of the curriculum.

Despite its successes, the new curricular model also presented notable challenges. Initially, there was a degree of resistance among faculty to transitioning away from lectures and adopting the active-learning format. One concern was that there would be a gradual disconnect from students regarding lecture content as the shift to asynchronous delivery eliminated both non-verbal and explicit feedback that instructors previously utilized to identify confusing concepts and refine their explanations and presentations. To help with this, surveys are available with every lecture to capture student feedback. Additionally, the active-learning sessions and exam questions help identify concepts that remain unclear after the lecture presentation. In these ways, lecture content can be updated appropriately, and more challenging concepts can be addressed during the in-person active-learning sessions. Another concern was adopting the active-learning format with which faculty had less experience. To help with this, the school provided many professional development sessions. The pharmacology team developed facilitator guides for each session to provide guidance regarding the flow of the session, areas that need extra emphasis or where additional questions for students could be beneficial, and expanded information as background for the facilitator. From a more global perspective, coordinating content across multiple campuses and disciplines occasionally led to timing misalignments, particularly when pharmacology sessions needed to integrate tightly with concurrent physiology, pathology, or clinical science instruction. Although repetition across sessions sometimes resulted in student frustration, these redundancies were pedagogically intentional and aligned with the principles of spiral reinforcement, even if the benefits were not always immediately evident to learners. Continued refinement of session sequencing and timing, along with coordination among Course Management Teams has helped mitigate these challenges; however, they remain inherent considerations in the ongoing administration of a complex, integrated curriculum.

## 5 Limitations

A limitation of this work is the inability to directly compare outcomes from the previous discipline-based pharmacology curriculum with those of the redesigned integrated curriculum. The curricular redesign involved multiple concurrent changes, including distributing pharmacology content across seven integrated courses rather than a stand-alone course, incorporating pharmacology questions within 23 of 24 organ-based examinations instead of using pharmacology-specific examinations, replacing locally-delivered lectures with standardized statewide recorded lectures, expanding active-learning instruction, standardizing instructional materials and assessments across nine campuses, and transitioning from an Honors/High Pass/Pass grading system to a Pass/Fail model. Because these changes were implemented simultaneously, it is not possible to isolate the effects of any single intervention or attribute observed outcomes to a specific component of the redesign. Rather than evaluating the impact of an individual curricular change, this study describes a comprehensive framework for integrating pharmacology within an organ-based curriculum and provides multiple indicators supporting its successful implementation. These indicators include preservation of dedicated pharmacology instructional time and faculty engagement, consistent delivery of curricular content across nine campuses, comparable student performance across campuses, and high levels of student satisfaction as reflected in both institutional and national survey data.

## 6 Conclusion

This study documents a successful decade-long transition from a traditional discipline-based model to an organ system-based curriculum within the pre-clerkship phase across nine geographically distributed campuses at Indiana University School of Medicine. Intentional curricular design and statewide pharmacology governance preserved the rigor and integrity of foundational pharmacology while expanding active-learning opportunities and maintaining high levels of student satisfaction, showing that large-scale curricular reform can be achieved without sacrificing disciplinary depth.

Additionally, the process yielded a replicable framework that may guide other regional or distributed medical schools seeking to align foundational science instruction with contemporary educational best practices while ensuring comparability across multiple campuses.

The sustained success of this transformation highlights the essential role of intentional curricular design paired with strong institutional leadership. Clear governance structures, consistent expectations, and collaborative course management allowed the integrated curriculum to maintain coherence across nine campuses while supporting local instructional strengths. The outcomes observed such as improvements in student satisfaction, robust pharmacology performance, and durable gains across multiple cohorts underscore that thoughtful planning, coordinated implementation, and ongoing refinement are critical to achieving meaningful and lasting curricular change.

## Data Availability

The original contributions presented in the study are included in the article/Supplementary Material, further inquiries can be directed to the corresponding author.
