# Supplementary material for: Standardizing pharmacology education in a systems-based curriculum: a longitudinal, multicampus innovations model
Source: Front Pharmacol. 2026 Aug 26;17:1911082. doi: 10.3389/fphar.2026.1911082 (PMC13553480; doi:10.3389/fphar.2026.1911082)
Supplement: Supplementary file 1 [file Table1.docx]

Supplementary Table 1

Share of total pharmacology hours and pharmacology active learning hours per course. FHD: Fundamentals of Health and Disease; HD: Host Defense, NB: Neuroscience and Behavior; CH: Cardiovascular and Hematology; RR: Renal and Respiratory; ERMD: Endocrine, Reproductive, Musculoskeletal, and Dermatologic; GIN: Gastrointestinal System and Nutrition

| Course | Total (h) | Active (h) | % Active | Share of total pharmacology hours (%) | Share of all pharmacology active learning (%) |
| --- | --- | --- | --- | --- | --- |
| FHD | 17.5 | 8.0 | 45.71 | 20.83 | 17.02 |
| HD | 6.5 | 2.0 | 30.77 | 7.74 | 4.26 |
| NB | 14.0 | 9.0 | 64.29 | 16.67 | 19.15 |
| CH | 13.0 | 7.0 | 53.85 | 15.48 | 14.89 |
| RR | 12.5 | 8.0 | 64.00 | 14.88 | 17.02 |
| ERMD | 12.0 | 7.0 | 58.33 | 14.29 | 14.89 |
| GIN | 8.5 | 6.0 | 70.59 | 10.12 | 12.77 |
